# Transcriptional-translational conflict: a novel exploitable tumor suppressive mechanism

**DOI:** 10.1038/s41392-023-01591-5

**Published:** 2023-09-25

**Authors:** Maria Mudryj, Paramita M. Ghosh

**Affiliations:** 1 VA Northern California Health Care System, Mather, CA USA; 2 University of California Davis Comprehensive Cancer Center, Sacramento, CA USA; 3 Department of Medical Microbiology and Immunology, University of California Davis, Davis, CA USA; 4 Department of Urologic Surgery, School of Medicine, University of California Davis, Sacramento, CA USA; 5 Department of Biochemistry and Molecular Medicine, School of Medicine, University of California Davis, Sacramento, CA USA

**Keywords:** Urological cancer, Cell biology

In a recent publication in *Cancer Cell*, Jana et al. uncover a phenomenon they call ‘transcriptional-translational conflict’ (TTC) where deletion of the ARID1A tumor suppressor elevated proliferation-associated transcripts, but up to 70% of upregulated mRNAs displayed no corresponding change in protein levels.^1^ The clinical implication of these findings is that patients with ARID1A-deficient bladder tumors may be susceptible to treatment with pharmacological inhibitors of protein synthesis.

Oncogenes and tumor suppressors have well defined roles in cancer—oncogenes like MYC and PIK3CA promote tumor growth while tumor suppressors such as PTEN and p53 prevent it. However, these genes often do not follow their supposed roles—loss of PTEN can upregulate TP53, promoting senescence, while MYC overexpression cause apoptosis through enhanced expression of BIM, and PIK3CA induce cellular differentiation via SH3RF1, an AKT substrate. Jana et al. now show a similar conflict in AT-rich interactive domain-containing protein 1 A (ARID1A), a SWI/SNF nucleosome remodeler mutated in ~25% urothelial malignancies.^1^ ARID1A is thought to be a tumor suppressor because its deletion upregulates transcription of oncogenic transcripts and promotes double stranded DNA (dsDNA) break repair, which results in tumor progression. However, knockout of *ARID1A* in mouse bladder urothelium did not increase the incidence of malignancies even though there was a deregulation of transcription resulting in an increase of cell cycle promoting mRNAs. Notably, this did not translate into an increase of cell cycle regulated proteins demonstrating a discordance between transcription and translation, which affected 70% of ARID1A transactivated mRNAs. The authors noticed that most of the transcripts subject to TTC had a high C/G content; previous reports support that high C/G content transcripts had a slower elongation rate,^2^ and they eventually concluded that this effect of ARID1A knockout on translation was due to a dampening of translation elongation caused by a decrease in active eEF2, a regulator of GTP-dependent ribosomal translocation.

The root cause of this elongation defect was decreased expression of the Ras superfamily guanine nucleotide exchange factor (GEF) RASGRP1, a direct target of ARID1A. RASGRP1 reduction was caused by deletion of ARID1A resulting in increased abundance of H3K27me3, indicative of elevated levels of the polycomb repressive complex 2 (PRC2) that restricts transcription (Fig. 1).^3^ RASGRP1 can initiate MAPK signaling; hence, a decrease in this signaling cascade culminated in activation of eEF2K, a highly conserved protein kinase that inactivates the eukaryotic elongation factor 2 (eEF2) via phosphorylation. Pharmachological targeting of EZH2, the catalytic component of PRC2, restored RASGRP1 protein levels and reduced eEF2 phosphorylation, thereby restoring protein synthesis rates.

**Fig. 1:**
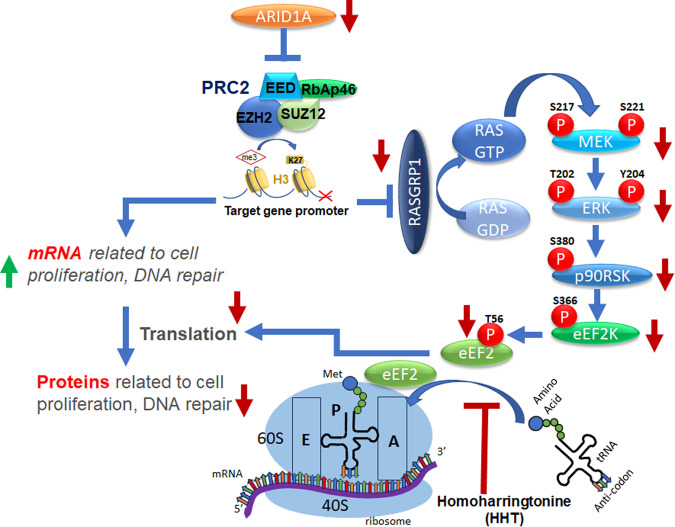
Schematic describing the work reported in *Cancer Cell*.^1^ The paper shows that the tumor suppressor ARID1A suppresses the expression of genes related to cell proliferation and DNA-damage repair; hence loss of this tumor suppressor increases the expression of these genes. However, the proteins related to the same genes are not equally upregulated because loss of ARID1A also suppresses RASGRP1, thereby decreasing the activity of the MAPK cascade, resulting in decreased phosphorylation of its downstream effector eEF2K. This also decreases phosphorylation of eEF2 at T56, resulting in a decrease in translation, which the authors call transcriptional-translational conflict (TTC)

ARID1A deletion compromised double strand DNA (dsDNA) break repair, excision repair, and repair of interstrand crosslinks, that significantly compromised DNA repair pathways and promoted genome instability. Protein levels of dsDNA repair genes, but not mRNA levels, were downregulated in ARID1A^fl/fl^ organoids, however, repression of eEF2 phosphorylation restored protein levels. In a classic model of bladder tumorigenesis, ARID1A deletion in N-butyl-N-(4-hydroxybutyl)nitrosoamine (BBN)-induced tumors^4^ caused an increase in γH2AX cleaved caspase-3, and DNA damage, compared to similarly treated WT-mice, although carcinogen exposure was comparable. Both WT and ARID1A^fl/fl^ mice developed the same number of tumors, however, ARID1A^fl/fl^ tumors were smaller than their WT counterparts.

In vivo modeling using the ARID1A/eEF2K double knockout tested the effects of relieving ARID1A deletion-mediated translation elongation deficiency, while retaining ARID1A deletion-dependent transcriptional transactivation, thus divorcing the two effects. Organoids from the urothelium of ARID1A knockout mice had limited replication potential but those from the ARID1A/eEF2K double knockout animals exhibited uncontrolled growth and elevated protein levels of ARID1A targets. Hence reinstating proficient translational elongation while maintaining ARID1A driven transactivation of pro-proliferative transcripts unveiled the oncogenic properties of ARID1A loss. BBN-induced ARID1A^fl/fl^ tumors showed 4-fold increase in eEF2 levels, but eEF2 phosphorylation increased only twofold, thereby enhancing translational elongation. ARID1A deletion in established tumors resulted in faster proliferating lesions with upregulated pro-proliferative mRNAs and upregulation of transcripts shown to increase RNA translation. Thus, TTC allows the suppression of oncogenesis even when a tumor suppressor is lost, by preventing protein synthesis despite an increase in the expression of oncogenic transcripts.

Can inhibiting protein synthesis in cells deficient for ARID1A accentuate TTC to suppress tumorigenesis? The translational inhibitor homoharringtonine (HHT) was used to treat bladder cancer organoids that were either ARID1A deficient or proficient. While HHT had minimal effects on ARID1A proficient cells, ARID1A deficient cells were sensitive to it. This analysis was recapitulated in preclinical studies using bladder cancer PDX models with low, medium, and high levels of ARID1A protein expression. In vivo tumor sensitivity to HHT inversely correlated with ARID1A levels suggesting that in the absence of ARID1A, pharmacological targeting of translational elongation can be utilized to restore its tumor suppressive properties.

The fundamental changes caused by ARID1A deletion are fueled by chromatin remodeling which ultimately results in altered expression of multiple transcripts. While cell cycle associated transcripts are upregulated by ARID1A loss, repression of RASGRP1 due to enhanced assembly of PRC2 at its promoter results in decreased translational elongation. Therefore, ARID1A loss alone is not sufficient to drive transformation but can, in conjunction with an increase in translational elongation, augment tumorigenesis. Reinstating the translational elongation block by pharmacological intervention restores TTC to limit tumorigenesis. Restoration of the elongation blockade would also be predicted to reduce levels of DNA repair proteins, which would hinder DNA repair and promote genome instability. A reduction of BRCA2-dependent dsDNA repair could be another therapeutic opportunity since these cells would be predicted to have heightened sensitivity to PARP inhibitors.^5^

This tumor suppressive mechanism in urothelial carcinoma is unique. While ARID1A is a positive regulator of translation, other tumor suppressors such as p53, PTEN, APC and PDCD4 are negative regulators of mRNA translation. However, since ARID1A is deleted or mutated in other malignancies as well, ARID1A may have a similar role in these tumors. Since the ARID1A-dependent transcriptome is likely to differ in different cellular contexts, the details of ARID1A-dependent tumor suppression or activation may be cell type specific. This study underscores that identifying changes in translation elongation is an underexplored facet of transformation that has a potential for discovery of novel therapeutic vulnerabilities.

## References

[1] Jana S (2023). Transcriptional-translational conflict is a barrier to cellular transformation and cancer progression. Cancer Cell.

[2] Bentele K, Saffert P, Rauscher R, Ignatova Z, Bluthgen N (2013). Efficient translation initiation dictates codon usage at gene start. Mol. Syst. Biol..

[3] Liu Y, Yang Q (2023). The roles of EZH2 in cancer and its inhibitors. Med. Oncol..

[4] Kelsey R (2018). Bladder cancer: BBN mouse model mimics human MIBC. Nat. Rev. Urol..

[5] Groelly FJ, Fawkes M, Dagg RA, Blackford AN, Tarsounas M (2023). Targeting DNA damage response pathways in cancer. Nat. Rev. Cancer.

